# Repeatability and Feasibility of Pressure Algometry for Quantifying Mechanical Nociceptive Threshold in the Thoracic Region of Calves

**DOI:** 10.3389/fvets.2020.00442

**Published:** 2020-07-31

**Authors:** Helen J. Williams, Jennifer S. Duncan, Dai H. Grove-White, Philippa J. Mahen, Amy V. Gillespie

**Affiliations:** ^1^Department of Livestock Health and Welfare, University of Liverpool, Liverpool, United Kingdom; ^2^Infection Biology, University of Liverpool, Liverpool, United Kingdom

**Keywords:** bovine, pressure algometer, pain, mechanical nociceptive threshold, inter-operator agreement, intra-operator agreement

## Abstract

Pressure algometry can be used to quantify mechanical nociceptive threshold (MNT) in humans and animals. If reliable this may be a useful tool to examine calves for increased mechanical sensitivity, which may be induced by disease or pain. This study measures the repeatability and feasibility of pressure algometry using a handheld digital pressure algometer (PRODPlus, Top Cat metrology) using three serial measurements applied to six sites on the thoraces of 35 healthy calves by two different operators. The range of MNTs recorded in healthy calves was 1.2–25 Newtons (median = 10.1 IQR = 7.1–14.0). A multivariable mixed effects model identified that the MNT's recorded were influenced by Operator, Site, and Calf. Intra and inter-operator reliability were measured by intra-class correlation coefficients (ICCs). Based on average ICCs, intra-operator reliability at two sites was good; one site overlying the ventral aspect of the 6th intercostal space [ICC = 0.79 95% CI (0.63–0.89)] and the other overlying the dorsal aspect of the 9th intercostal space [ICC = 0.75 95% CI (0.56–0.87)]. Average ICCs for three other measurement sites were moderate or poor, and one site proved unfeasible. For inter-operator agreement average ICCs showed that agreement was also good at the same 6 and 9th intercostal space, [ICCs = 0.77 95% CI (0.35–0.90) and 0.77 95% CI (0.54–0.88), respectively], agreement was moderate for the remainder of the sites. This study identifies two sites that are potentially useful for monitoring of thoracic sensitivity as an indicator of pain in calves by means of pressure algometry using the average of three measurements. It also identifies sources of variability to be considered when applying the tool for clinical or research purposes.

## Introduction

Freedom from pain, injury, and disease is one of the Farm Animal Welfare Council's (FAWC) Five Freedoms and a central tenet for safeguarding the welfare of farmed animals. To ensure optimization of this freedom it is necessary to prevent, rapidly diagnosis and treat conditions which cause pain, injury, or disease in farm animals (1).

Bovine respiratory disease (BRD) is a leading cause of morbidity and mortality of dairy and beef cattle worldwide and has substantial impact on animal welfare and economics (2). Non-steroidal anti-inflammatory drugs (NSAIDs) are recommended as an adjunct to antibiotic therapy for treatment and have been shown to be beneficial in terms of live weight gain (3), although pain associated with BRD has not yet been objectively quantified.

Accurate measurement of pain associated with BRD is essential to fully determine the welfare impact of BRD on cattle, quantify the potential benefits to animal welfare of pain alleviation, and enable consistent, evidence based, analgesic protocols to be developed for the condition. Therefore, a valid, reliable, and feasible method for the measurement of pain associated with BRD in cattle is required.

The experience of pain cannot be directly measured in animals, but can be inferred using for example physiological, behavioral, and performance indicators (4). These are used individually or in combination to improve test sensitivity and specificity (5). Physiological indicators such as measurement of cortisol, or inflammatory mediators require invasive sampling and laboratory assessment. In addition, there are issues around the specificity of measures for pain as distinct from stress or inflammation. Therefore, physiological measures can have practical limitations for on farm measurement of pain in cattle. Performance measures, for example live weight gain can be useful, but these are long term measures of welfare impact, rather than short term assessment of the animal's direct experience of pain (6). Behavioral indicators of pain have been widely used as they are generally observational, non-invasive, low cost, and practical for field use. Most cases where behavioral responses have been used to indicate pain are diseases or procedures where the pain is likely to be relatively high. Examples include the degree of head shaking and ear flicking following dehorning (7) or locomotion scoring, which describes the degree of lameness in cattle based on behavioral postural changes (8). More recently, it has been demonstrated that subtle behavioral changes such as ear position and facial expression may be used to assess pain in cattle for a number of conditions (9). The principle disadvantage of behavioral measures of pain is the subjective assessment technique of different operators which may affect test performance (4).

Pressure algometry is an objective, behavioral, calibrated, short-term indicator of increased sensitivity indicative of pain, used in research and clinical practice in humans and animals. A pressure algometer measures the force applied to tissues via a probe, which is referred to as a noxious stimulus. In humans, the pain pressure threshold (PPT) is the lowest pressure at which the patient verbally reports perceiving pain (10). An increase in sensitivity to this noxious stimulus may correspond to increased sensitivity of nociceptors at the test site and is interpreted as increased sensitivity at the test site. Since animals are unable to state when they feel pain the mechanical nociceptive threshold (MNT) is recorded instead of the PPT, this is the amount of pressure needed to produce a pre-determined behavioral response indicative of pain (11). The use and experience of this tool in people lends additional validity to its interpretation as an indicator of pain in animals. The algometer measures the pressure applied to tissues and the response of the human/animal is recorded. The response would typically be vocalization (verbal acknowledgment of the pain experienced in humans), avoidance and defense behaviors (withdrawal reflex, moving away from the stimuli).

Pressure algometry has been used to assess pain and effectiveness of analgesia for research purposes in a range of farm animal species, including pigs (11) and sheep (12). In cattle, it has been used to assess pain sensitivity in dairy cattle with mastitis (13), lameness (14–16); and following dehorning (7).

Pressure algometry has been shown to have good inter-operator repeatability when carried out on the limbs of normal dairy cattle using the mean of several tests and consistent test sites (17). Pressure algometry is an objective, repeatable and non-invasive, short term indicator of pain, and therefore may offer a practical method of assessing pain associated with BRD in cattle on farms. As the method has not been previously applied for BRD, the aim of this study was to assess its repeatability and feasibility when applied to the thorax of healthy calves; the anatomical site where the underlying pathology caused by BRD is found.

## Materials and Methods

This study design was approved by the University of Liverpool Veterinary Research Ethics Committee (reference VREC 369). Research was carried out under Project License PPL708757 issued by the UK Home Office under the Animals (Scientific Procedures) Act 1986.

### Case Selection and Randomization

Thirty-six healthy Holstein Friesian dairy heifer calves between 2 and 12 weeks of age were enrolled from a convenience sample of two commercial dairy herds in northwest England. Calculations of sample size were complicated by the lack of pre-existing data for an exactly comparable situation. A previous study suggests standard deviations for a single observer to be ~25% of the total range of the algometer, 0–25 N in this instance (17). Subsequently, it was calculated that 36 calves were required in order to detect a difference of 5 N or more between operators.

Calves were housed in small groups of 4–6 animals in straw-bedded pens. Calves were fed according to normal husbandry practices for each commercial farm. Briefly, diet was 3L of milk replacer fed twice daily, calf-rearer pellet, ad lib forage, and ad lib water. Typically calves older than 8 weeks would no longer be fed milk replacer. As all calves were hand reared, they were habituated to handling by farm staff, but were not habituated to the researchers. Farm visits were carried out in the middle of the day, approximately halfway between milk feeds. A convenience sample of calves were examined at each visit. Each calf was restrained using a halter and examined by a veterinary surgeon. The examination included taking the rectal temperature, assessment of umbilicus, and joints for swelling, fecal score, and auscultation of the thorax to detect heart or lung abnormalities. Only calves that had no abnormalities on clinical examination were considered for inclusion in the study. These calves were respiratory scored according to the Wisconsin calf respiratory scoring chart (18). Calves scoring zero, or 1–2 where the positive score resulted only from a rectal temperature >38.3°C but <39.0°C, were enrolled into the study.

Calves not eligible for inclusion in the study but with a respiratory score <5 and otherwise clinically normal (as per McGuirk and Peek, 2014) were released. Calves scoring five or more were not eligible for inclusion and were released for treatment as per normal practice on that farm. Seventy-three calves were excluded from the study.

Once selected for inclusion in the study, the experimental procedure lasted approximately half an hour per calf and was carried out with the calf in its “home” pen to avoid any effect on results that may be caused by a novel environment. Although calves were not specifically separated from their group, researchers ensured other calves did not interfere with the testing procedure. Two experienced cattle veterinary surgeons performed the pressure algometry. Calves were randomized in blocks of four according to whether testing was carried out on the left or right side of the chest and whether operator A or operator B went first. The four permutations (Left side of chest, operator A first; Left side, B first; Right side, A first; Right side, B first) were recorded on slips of paper and placed in an envelope. Once a calf had been deemed suitable for enrolment, a slip of paper was selected, and the calf was assigned to that group.

### Site Selection and Identification, and Order of Testing

**Figure 1:**
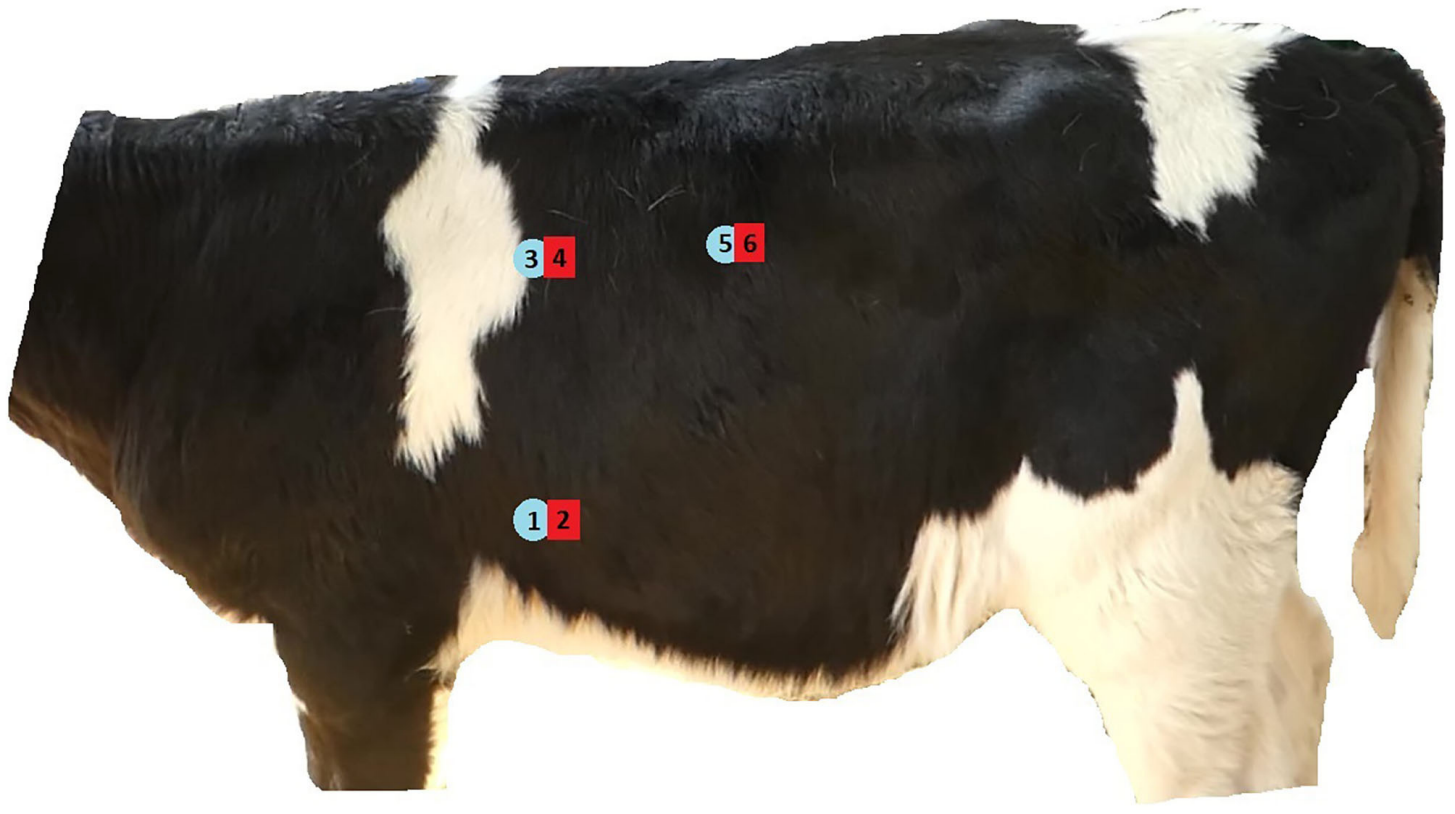
Illustration showing approximate location of six sites used to test repeatability and feasibility of pressure algometry for measuring mechanical nociceptive threshold on calves' thoraces.

Mechanical nociceptive threshold testing was carried out at six sites on either the left-hand or right-hand side of the chest, selected to compare the reliability over different lung fields and between testing sites overlying a rib compared to those overlying an intercostal space. The sites were as follows (Figure 1):

1. Over the 6th rib ~5 cm dorsal to the costo-chondral junction.
2. Over the 6th intercostal space level with site 1.
3. Over the 6th rib over the most dorsal 5 cm of rib which could be palpated.
4. Over the 6th intercostal space level with site 3.
5. Over the 9th rib ~5 cm below the most dorsal point at which the rib could be palpated.
6. Over the 9th intercostal space level with site 5.

Each site was marked prior to the procedure by clipping a small patch of overlying hair with scissors, to ensure both operators made measurements at the same site on each testing occasion.

### Use of the Algometer

A handheld digital pressure algometer (PRODPlus, Top Cat metrology, Cambridgeshire, UK) accurate within a force range of 0.5–25 N and a tip with a diameter 4 mm was used. The tip was chosen based on clinical experience and to avoid tissue damage. The algometer was calibrated and the rate of force application was set by the manufacturer at 2 N/s. This could be monitored by the operator using the red and green lights on the algometer indicating if force needed to be applied faster or slower. Before use on an animal each operator practiced using the pressure algometer on an inanimate object until they could control the rate of force application reliably. A cardboard canopy was taped around the screen so the operator could not see the force being applied but it could be monitored by an assistant.

Before each use the algometer was reset. Prior to the first application the operator placed a hand over the thorax at the test site and applied light pressure to avoid startling the calf when the pressure algometer was first applied. When the calf was settled the hand was replaced by the pressure algometer. Force was applied perpendicular to the thorax at a rate of 2 N/s until the calf demonstrated an avoidance reaction, either by moving away, kicking, or a sharp movement of the head. As soon as an avoidance reaction was noticed by the operator the pressure algometer was removed. To minimize bias, the operator did not view the screen of the algometer until after the test was complete. If there was no avoidance reaction once the force reached 25 N (the upper limit of the accurate force range), as observed by an assistant, the test was stopped to avoid any damage to soft tissues. This procedure was repeated three times at the first site by the first operator with a 30 s gap between each application. Then after a 30 s gap, the second operator carried out the procedure three times in the same manner.

The procedure was then repeated for each of the measurement sites in sequential order with the same order of operators.

If the calf became unsettled during the procedure and would not stand without restraint that would be likely to impede voluntary movement, then the calf would firstly be moved for example to the other side of the pen, and if it still would not stand the procedure was abandoned.

### Data Analysis

All data were entered onto a spreadsheet (Excel 2010, Microsoft) and then imported into Stata 15 for analysis (Statacorp).

The number of occasions where test results were excluded due to the algometer slipping off the correct site was recorded in addition to the mechanical nociceptive threshold (MNT) at each site. The data regarding whether the probe slipped were categorized according to whether the test site was over a rib or over an intercostal space. The relative risk ratio for the algometer slipping off a test site over a rib compared to over an intercostal space was calculated and a Fisher's exact test was used to determine significance.

A multivariable mixed effects model which accounted for censoring was implemented in Stata 15 using the metobit function with MNT as the outcome. Side of thorax, site, operator and test number (whether the measurement was taken at the first, second, or third application of the pressure algometer) were initially included as independent categorical variables; and age of calf in days was included as an independent continuous variable. It was considered possible that readings for MNT could be clustered within operator, however since there were only two operators we cannot report an overall operator effect that is applicable to a population of operators, therefore operator was considered as a fixed effect. The model also included calf identity as a random effect. A backwards stepwise model building strategy using likelihood ratio testing was employed to determine which variables would remain in the final model. Variables with *P* < 0.1 in the initial model were considered for inclusion in the final model.

Intra-operator reliability between the measurements taken at each site, and inter-operator reliability between each operator's mean of the three measurements taken at each site, were assessed using intra-class correlation coefficients (ICCs). For both, ICC estimates and their 95% confident intervals were calculated based on absolute-agreement using 2-way mixed-effects models. Both individual and average ICC's are reported. For intra-operator reliability *k* = 3, individual ICCs indicate the reliability between each of the individual tests on one calf whereas the average ICC's indicate the reliability of the mean of the three tests in each calf compared to all other calves. For inter-operator reliability *k* = 2, individual ICCs indicate the reliability between the two operators when testing the same calf at the same site, whereas average ICC indicates the reliability between operators for all calves at the same site.

Intra-class correlation coefficient values <0.5 were considered to be indicative of poor reliability, from 0.5 to 0.74 moderate reliability, from 0.75 to 0.9 good reliability, and >0.9 excellent reliability (19).

## Results

**Figure 2:**
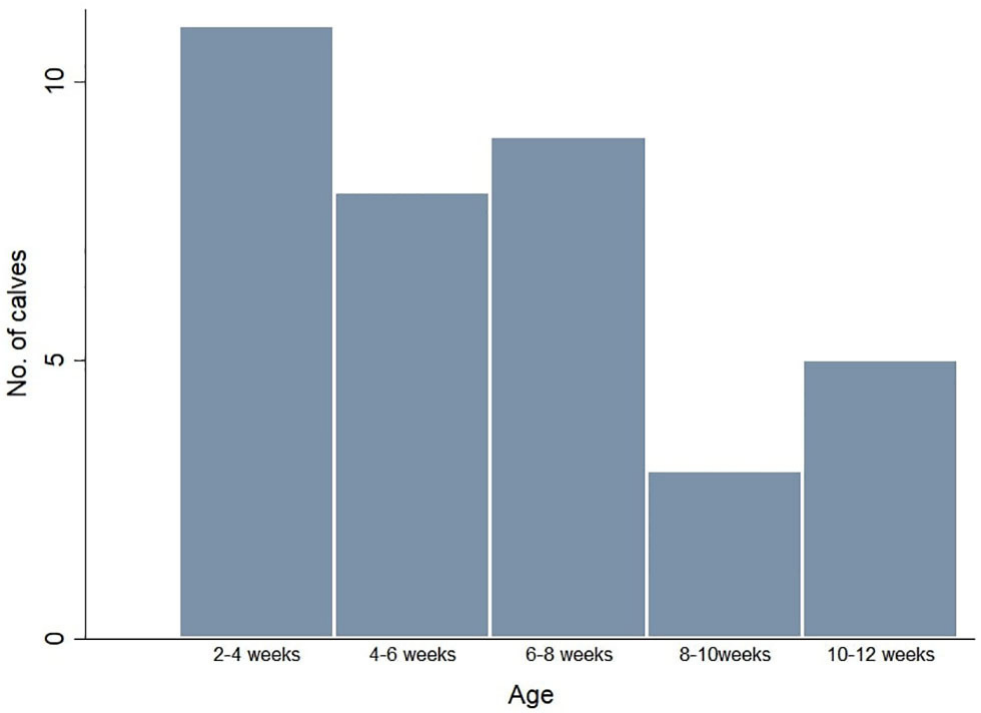
Distribution of ages of calves included in the study.

Thirty-six calves were initially enrolled in the study. The median age of the calves was 41 days (Interquartile range = 25.5–55 days) (Figure 2). One calf was excluded as its constant movement meant it was not possible to obtain readings.

### Feasibility of Pressure Algometry

It was apparent after carrying out pressure algometry on five calves that it was problematic obtaining readings at site 5 (over the dorsal aspect of the 9th rib, Figure 1). Due to the rounded nature of the rib at this site the operators found it impossible to apply force without the tip of the algometer slipping off the rib into the intercostal space. Therefore, any readings collected at this site were excluded on grounds of feasibility from further analysis and no further pressure algometry was carried out at this site.

In total, the pressure algometer slipped off the remaining test sites on 62/1050 (5.9%) occasions, and any readings collected in these instances were excluded from further analysis.

The greatest number of readings that were inadmissible for this reason was at site 1 where 26/105 (24.8%) of the readings taken by operator A and 13/105 (12.4%) of the readings taken by operator B slipped off the intended site (Table 1). There were significantly more exclusions for slipping on sites located over a rib (sites 1 and 3). The relative risk for at least one test per calf carried out over a rib slipping was 0.69; whereas the relative risk of slipping when testing over an intercostal space was 0.09. Therefore, the risk ratio for slipping when testing over a rib compared to over an intercostal space was 8.0 (*P* < 0.001).

**Table 1:** MNT readings taken from the thoraces of calves that exceeded the 25 N upper limit of the pressure range used in this study, or that were excluded for probe slippage during testing.

**Site**	**Operator A**		**Operator B**	
	**No. of readings excluded for slipping**	**No. of readings >25 N**	**No. of readings excluded for slipping**	**No. of readings >25 N**
1	26 (24.8%)	1 (1.0%)	13 (12.4%)	2 (1.9%)
2	0	0	1 (1.0%)	0
3	16 (15.2%)	3 (2.9%)	3 (2.9%)	1 (1.0%)
4	2 (1.9%)	5 (4.8%)	1 (1.0%)	2 (1.9%)
6	0	3 (2.9%)	0	3 (2.9%)

*Findings are displayed according to which operator carried out the test and which sampling site they occurred at*.

On 20/1050 (1.9%) of occasions the force applied reached 25 N. The pressure algometer was removed at this point, the reading was recorded as 25.1 N and included in further analysis (Table 1).

Calves reacted to the pressure algometer in different ways. For example, some calves reacted to the pressure by lifting a leg (either fore-limb or hind-limb), whilst others turned or lifted their head or moved away. Videos 1–3 in the Supplementary Materials illustrate these three variations.

### Range of MNT Values in Healthy Calves

**Figure 3:**
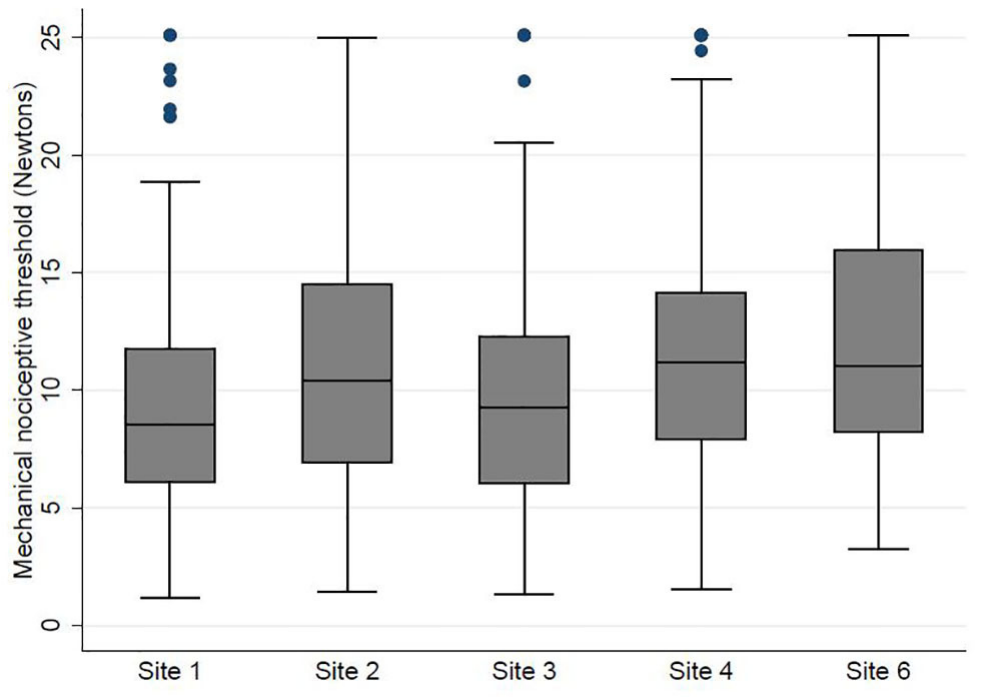
A Tukey boxplot displaying mechanical nociceptive threshold at five sites of the thorax of 35 calves measured by pressure algometry.

After exclusions there were 988 MNT readings. The mechanical nociceptive threshold (MNT) ranged from 1.2 N to >25 N (median = 10.1 IQR = 7.1–14.0) (Figure 3).

A metobit model was fitted with MNT as the outcome, after backwards stepwise elimination, operator, site, and calf identity remained as significant variables affecting MNT. Sites 2, 4, and 6 (sites overlying intercostal spaces) were associated with higher MNT readings than sites 1 and 3 (sites overlying ribs). Operator B recorded lower MNT's compared to operator A (Table 2).

**Table 2:** Results from a multivariable mixed effects model accounting for censoring (metobit), showing that the site at which the pressure algometer was applied, the operator and calf identity significantly affected the MNT measured over the thoraces of calves.

**Variable**	**Coefficient**	**95% CI**	***P*-value**
Site 1	Reference		
Site 2	1.47	0.52–2.41	0.002
Site 3	0.18	−0.78–1.15	0.709
Site 4	2.08	1.13–3.03	<0.0001
Site 6	2.84	1.89–3.78	<0.0001
Operator B compared to A	−1.36	−1.95–0.78	<0.0001

*Calf identity variance estimates = 5.41 (95% CI = 3.17–9.24) P < 0.001*.

*Intraclass correlation coefficient = 0.198 (95%CI = 0.13–0.30)*.

### Reliability of MNT Values in Healthy Calves

Reliability within operator between the measurements taken at each site was assessed using intra-class correlation coefficients (Table 3). When considering the individual ICCs, a moderate correlation was demonstrated by operator B at sites two and six of 0.56 (95% CI 0.36–0.73) and 0.50 (95% CI 0.3–0.68), respectively; however, all other individual ICCs were poor indicating that the three measurements were inconsistent. The average ICCs were generally higher, the highest reliabilities were recorded by operator B at sites two and six (0.79 95% CI 0.63–0.89 and 0.75 95% CI 0.56–0.87) which can be considered good, all other ICCs were moderate or poor (Table 3).

**Table 3:** Intra-operator reliability for MNT measured using pressure algometry over the thoraces of calves.

**Site**	**Operator**	**Number of calves included**	**Individual ICC (95% CI)**	**Average ICC (95% CI)**	**Prob > F**
1	A	18	0.29 (0.24–0.60)	0.56 (0.07–0.81)	0.017
1	B	25	0.43 (0.18–0.67)	0.70 (0.40–0.86)	<0.001
2	A	35	0.48 (0.28–0.67)	0.73 (0.54–0.86)	<0.001
2	B	34	0.56 (0.36–0.73)	0.79 (0.63–0.89)	<0.001
3	A	24	0.39 (0.13–0.64)	0.65 (0.31–0.84)	0.001
3	B	32	0.22 (0.02–0.45)	0.45 (0.05–0.71)	0.016
4	A	33	0.48 (0.28–0.67)	0.74 (0.54–0.86)	<0.001
4	B	34	0.17 (−0.02–0.41)	0.39 (−0.06–0.67)	0.042
6	A	35	0.48 (0.28–0.67)	0.74 (0.54–0.86)	<0.001
6	B	35	0.50 (0.30–0.68)	0.75 (0.56–0.87)	<0.001

*Individual ICC shows reliability between each of the individual tests on one calf; and Average ICC shows the reliability of the mean of the three tests in each calf compared to all other calves. Where the algometer had slipped off the test site on one test the calf was excluded from analysis for the relevant operator and site*.

Inter-operator reliability was assessed using ICCs carried out on the mean of the three measurements taken at each site. Individual ICCs showed correlation between operators was moderate or poor at all sites when comparing measurements taken from a single calf. However, when considering data from all calves, average ICCs were good at sites two and six: 0.77 (95% CI 0.35–0.90) and 0.77(95% CI 0.54–0.88), respectively (Table 4).

**Table 4:** Inter-operator agreement for mean MNT calculated from three serial measurements using pressure algometry over the thoraces of calves.

**Site**	**Number of calves**	**Individual ICC (95% CI)**	**Average ICC (95% CI)**	**Prob > F**
1	14	0.54 (0.08–0.82)	0.70 (0.14–0.90)	0.010
2	34	0.63 (0.21–0.83)	0.77 (0.35–0.90)	<0.001
3	21	0.39 (−0.05–0.70)	0.56 (−0.10–0.82)	0.039
4	32	0.40 (0.04–0.66)	0.57 (0.07–0.79)	0.002
6	35	0.62 (0.37–0.79)	0.77 (0.54–0.88)	<0.001

*Individual ICC shows agreement between operators when testing the same calf at the same site. Average ICC shows the agreement between operators for all calves at the same site. Where the algometer had slipped off the test site on one test the calf was excluded from analysis for the relevant site*.

## Discussion

This study identified two sites (site 2- over the 6th intercostal space and site 6- over the 9th intercostal space, Figure 1) on a calf's thorax that may be suitable for indicating thoracic pain sensitivity by taking three serial measurements using pressure algometry. The study also identified sources of variation in measurements (calf and operator) that should be considered before using this procedure for clinical or research purposes. Therefore, if comparisons of tests were made within calf (for example before and after an intervention), at sites 2 and 6, and a single operator performed all tests, uncertainty around measurement error could be minimized.

### Feasibility of Pressure Algometry in Calves

There was unexpected difficulty in carrying out the testing at sites 1, 3, and 5 which overlay a rib. Operators found that the probe could slip off the rib making it impossible to apply even pressure when this occurred. It is possible that exclusion of this data reduced study power for sites 1 and 3 and contributed to the reduced reliability we found for the method at these sites. The problem was most pronounced at site 5 over the 9th rib which had to be excluded all together. This was thought to be due to the rib surface being more curved than the 6th rib (sites 1 and 3). The problem of the probe slipping may be less pronounced in older calves and adults where the ribs are larger, and different probe sizes may also perform differently. However, this problem would preclude sites overlying a rib in calves under 12 weeks of age being utilized for pressure algometry using the same hand-held algometer with the same probe in future studies or clinical work. A small number of readings (1.9%) reached the maximum limit of the algometer's accurate range, meaning that data had to be right censored in these cases. It was unclear whether these high values occurred due to observers missing calf responses, or whether responses were truly absent.

### Range of MNT Values in Healthy Calves

Operators found that there was wide variation in calf behavior and reactions. Some calves showed an obvious avoidance reaction while in other cases it was more subtle. A range of avoidance reactions was demonstrated including a head turn or leg lift. This variation between calves was demonstrated by the model results which showed 19.8% of variation was attributable to the calf identity. It was unclear whether this difference was truly due to difference in sensitivity threshold; or resulted from behavioral differences or operator technique. It may be that testing other body sites where a more definite criteria for a response could be set would yield more reliable results, for example considering a leg lift as an endpoint when testing limbs (16) or a head movement when testing horn buds (7). Operator and site were also factors affecting the range of MNT values measured. Operator B recorded significantly lower MNT than operator A, −1.36 newtons 95% CI (−1.95- −0.78). In common with a previous study using algometry (17), we found inter-observer differences indicating that for comparative testing, a single operator should be used. The time taken to apply a single test is a matter of a few seconds as pressure is increased by 2 N/s. Small differences in operator reaction times would be detected in the MNT data and could explain the variation between operators.

Previous studies have demonstrated that MNT varies depending on the site of the body used. For example MNT is greater when measured on the thoracic limbs of pigs as opposed to the pelvic limbs and the lateral metacarpi/tarsi compared to the dorsal (20). There have been similar findings in cattle where the MNT was significantly higher on the lateral aspect of the limb compared to the dorsal aspect (17). It has been speculated that higher MNT's are observed where there is more soft tissue coverage (17, 21). This is consistent with the findings of this study where MNT's were significantly higher in intercostal spaces (sites 2, 4, and 6) compared to those measured at sites 1 and 3 which overlay a rib (Table 2).

### Reliability of MNT Values in Healthy Calves

Intra-operator reliability between the three measurements at a single site on a single calf was generally poor (ICC <0.5) (Table 3). However, inter-operator reliability between the mean of the three measurements at a single site were generally better, with moderate agreement at site 1 and good agreement at sites 2 and 6 (Table 4), indicating that a mean of three measurements improves consistency over a single measurement. This was in agreement with a previous study using a pressure algometer on the legs of dairy cattle (17).

Intra-operator reliability (Table 3) showed variation between operators A and B and the sites used. Operator B was more reliable at measuring MNT at sites 1, 2, and 6, whilst operator A was more reliable at sites 3 and 4. These findings agree with MNT modeling data (Table 2) confirming that operator does influence the MNT. Testing at sites 2 and 6 had the best intra- and inter-operator reliability. These testing sites overlying intercostal spaces also resulted in fewer exclusions due to the probe slipping. Therefore, these sites are likely the best candidates for further application.

This study was conducted using only heifers as algometry studies in people have demonstrated gender differences in the response (22). The age range was restricted to avoid the neonatal period as it is recognized that human neonates differ in their sensitivity to noxious stimulation (23). It is unknown whether similar gender or age differences exist in cattle and further work would be needed to determine this.

In conclusion, in the healthy animal pressure algometry can be a reliable tool for measuring MNT on calves' thoraces when applied by a single operator. Reliability of MNT is improved by using an average of three measurements, and by using sites 2 and 6. Further work should apply the tool in calves affected by BRD to investigate changes in MNT indicative of sensory changes induced by pain or disease.

## Data Availability Statement

The datasets generated for this study are available on request to the corresponding author.

## Ethics Statement

The animal study was reviewed and approved by the University of Liverpool Veterinary Research Ethics Committee (reference VREC 369). Research was carried out under Project License PPL708757 issued by the UK Home Office under the Animals (Scientific Procedures) Act 1986. Written informed consent was obtained from the owners for the participation of their animals in this study.

## Author Contributions

HW, AG, and JD contributed conception and design of the study. HW and AG performed the statistical analyses and wrote the first draft of the manuscript. DG-W was consulted for revision of statistical analyses. All authors were involved in conducting the study, collecting the data, contributed to manuscript revision, read, and approved the submitted version.

## Conflict of Interest

The authors declare that the research was conducted in the absence of any commercial or financial relationships that could be construed as a potential conflict of interest.
